# Self-Adhesive Mask and Homemade Carbon Ink: Turning Unusable Screen-Printed Electrodes into a New Voltammetric Sensor

**DOI:** 10.1021/acsomega.5c11328

**Published:** 2026-01-02

**Authors:** Gabriel Chitolina-Rodrigues, Adriano Rogerio Silva Lima, Duane Bortot, Caio Raphael Vanoni, Cristiane Luisa Jost, Habdias de Araujo Silva-Neto

**Affiliations:** Departamento de Química, Universidade Federal de Santa Catarina, Campus Universitário Trindade, 88040-900 Florianópolis, SC, Brazil

## Abstract

Screen-printed carbon electrodes (SPCEs) are widely employed in sensing due to their low cost, especially the model DRP-110. However, their disposable nature limits their long-term applications. In this work, we present a sustainable methodology for recovering the electrode model DRP-110-U75. Unusable SPCEs were made usable again after employing a stencil-printing approach. After recuperating the electrodes (Re-SPCE), characterization experiments upon the Re-SPCEs have indicated similar morphological aspects and catalytic ability when compared to as-purchased SPCEs. As a proof of concept, voltammetric analysis of dopamine (DOP) in artificial urine and tryptophan (TRP) in supplement capsules was successfully employed, exhibiting limits of detection of 2.15 and 0.2 μmol L^–1^ for DOP and TRP, respectively. These results confirm that the proposed method can extend the life cycle of SPCEs.

## Graphical abstract

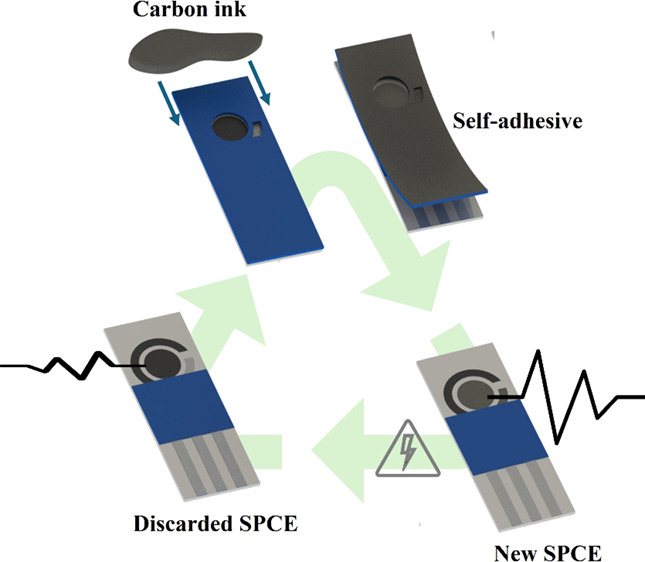

## 1 Introduction

Screen-printed carbon electrodes (SPCE) have been developed and improved by research groups since the 1990s, enabling an increase in their use in the current commercial research industry.^1^ According to Scopus, from 2000 to 2024, the use of SPCEs as sensing materials has increased substantially. In the past year (2024), the number of papers employing SPCEs exceeded 200 documents, which represents 20-fold better when compared to the number of publications from 2010 (Figure S1; available in electronic Supporting Information; ESI). As a widely known example, which accounts for approximately 5% of the population, point-of-care testing for glucose in humans is based on a modified SPCE device that helps control diabetes, with a worldwide marketing strategy that generates billions of dollars in revenue.^2^

SPCEs are composed of conductive inks made from a mixture of carbon allotrope and a polymeric binder, which are deposited onto a predesigned substrate such as ceramic, paper, or plastic.^3,4^ One popular example of SPCE is the commercially available DRP-110 model.^5^ Although SPEs are manufactured as single-use devices and are typically discarded after a few measurements, some practices can prolong their functional lifespan. For instance, avoiding highly adsorptive analytes and limiting scans at high potentials may reduce the level of surface degradation. Mechanical polishing procedures are generally unsuitable for SPCEs due to the low thickness of the printed ink layer and its relatively low hardness.^6^ Nevertheless, several studies have explored different surface pretreatment approaches to enhance performance and increase electrode durability. Such strategies are more commonly applied to gold and platinum screen-printed electrodes, where physical and chemical pretreatments are justified by the high cost of gold and the significant hardness and chemical stability of platinum.^7,8^

Due to their interesting analytical characteristics, SPCEs have been applied to different situations, such as pesticide detection,^9–12^ biosensing,^13–15^ heavy metal analysis,^16–19^ food additives,^20–22^ and neurotransmitters.^23–26^ Some reported examples include the detection of parathion, glucose, Cd, Pb, ascorbic acid, caffeine, dopamine (DOP), and tryptophan (TRP).^27^

Despite their analytical advantages, the cost of commercial SPCEs can remain a limiting factor, especially for sensing experiments which require large numbers of disposable sensors and/or for laboratories operating under budget constraints.^28–30^ A commercial SPCE, such as the model DRP-110, is priced at ∼USD 4.50 per unit, which can substantially increase the operational cost of routine analyses. If SPCEs are reutilized, their analytical applicability can be drastically improved, mainly in developing regions. However, there are no reported studies focused on recovering SPCEs, especially the model DRP-110, after being considered unusable. This alternative of recycling materials can bring a new exploration of SPCEs and contribute to the principles of the circular economy.

This work describes, for the first time, the reuse of commercial SPCEs, aiming to perform electrochemical measurements of DOP and TRP. Unusable SPCEs were collected and turned great again after employing the stencil-printing approach. To cover the reference and working electrodes again, a glass-vintage-based graphite conductive ink was used. The proposed electrodes were carefully characterized in terms of morphological and electrochemical aspects, and their catalytic ability was compared with that of as-purchased SPCEs. As a proof of concept, the Re-SPCEs were utilized for detecting DOP and TRP in artificial urine and food supplements, respectively. Also, the ecological impact of the proposed analytical methodologies was evaluated.

## 2 Experimental Section

### 2.1 Chemicals and Materials

DOP, TRP, potassium hexacyanoferrate­(II) trihydrate, potassium hexacyanoferrate­(III), graphite powder (Ø = 50 μm), and Surine were purchased from Sigma-Aldrich (St. Louis, MO, USA). Boric acid was purchased from Synth (Diadema, SP, and BR). Acetic acid, phosphoric acid, sodium phosphate dibasic heptahydrate, and sodium phosphate monobasic monohydrate were obtained from Vetec (Rio de Janeiro, RJ, BR). All aqueous solutions were prepared with ultrapure water supplied by a Millipore Milli-Q system (Bedford, MA, USA), with a resistivity of 18.2 MΩ cm. A glass varnish binder and self-adhesive sheet (thickness: 125 μm) were obtained from Acrilex (São Bernardo do Campo, SP, BR) and Imprimax (São Paulo, SP, Brazil), respectively.

### 2.2 Instrumentation

Electrochemical measurements were performed using a PalmSens electrochemical workstation (Vleugelboot, CL, NL) controlled by PSTrace software, version 5.11. To perform the comparative experiments, an SPCE model DRP-110-U75 (Ag as reference (RE), carbon as auxiliary (AE), and carbon as working (WE) electrodes, Ø = 4 mm) was purchased from Metrohm (Herisau, AR, NL). For manufacturing stencil masks, a craft cutter printer model Cameo 4, obtained from Silhouette (Belo Horizonte, MG, and BR), was used.

### 2.3 Collection and Recovery Process of SPCE

A homemade self-adhesive mask and carbon-based ink were utilized to recycle SPCEs. SPCEs, model DRP-110-U75, were collected at local research laboratories after being considered unusable. A total of ∼100 different devices were stored over the past year. Before the homemade mask was manufactured, a similar layout of the SPCE was modeled by employing the Silhouette Studio software. That stencil mask was projected, aiming to reprint two different regions of the SPCE, the WE and RE. The self-adhesive mask was fixed on the SPCE surface, covering all regions of the SPCE except the WE and RE. For preparing the conductive ink, 1.5 g of graphite powder and 1.5 g of glass varnish were mixed in 4.0 mL of acetone for 10 min at ∼500 rpm.^31^ Next, the prepared carbon-based ink was dispersed through the stencil mask/SPCE by using a spatula. After 60 min, the stencil mask was removed, and the device was ready to be reutilized. The proposed protocol for recovering the SPCE can be observed in Figure 1 and Video S1 (please, see it in the ESI).

**Figure 1:**
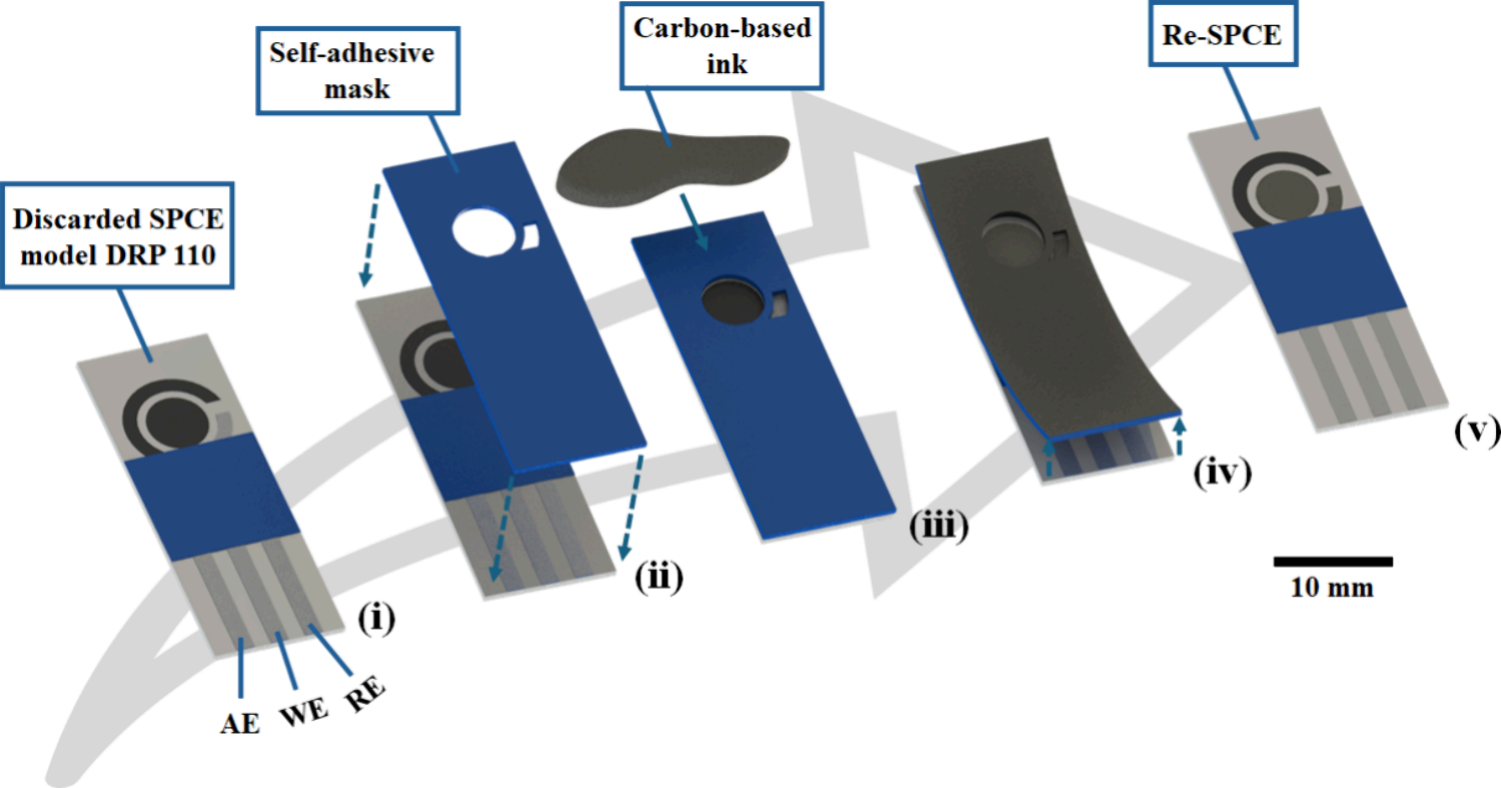
Schematic representation of the SPCE recovery process: (i) collection of discarded SPCEs; (ii) cutting of the self-adhesive stencil mask according to the electrode dimensions and attachment onto the device surface; (iii) deposition of the conductive ink; (iv) removal of the self-adhesive mask; and (v) Re-SPCE ready for use.

### 2.4 Electrochemical Measurements

For conducting the selection of the RE and evaluating the repeatability and reproducibility studies of the Re-SPCEs, the cyclic voltammetry (CV) technique was used. For selecting an adequate RE to use in the proposed electrochemical cell, three different options were evaluated: (i) a saturated Ag/AgCl model RE-1B, (ii) an as-purchased RE from the SPCE, and (iii) a recovered RE coated with conductive carbon ink. In all cases, the WEs were also covered with conductive carbon ink. Repeatability tests were carried out by performing 10 consecutive measurements using Re-SPCEs. As a comparative study, the same experiment mentioned above was also conducted using an as-purchased SPCE (model DRP-110). For the reproducibility experiment, CVs were recorded by employing 16 different sensors. In this experiment, freshly discarded SPCEs and Re-SPCEs were employed. All abovementioned measurements were carried out in 1.0 mmol L^–1^ [Fe­(CN) _6_]^3–/4–^ prepared in 0.1 mol L^–1^ KCl within the potential range of −0.5 to 0.8 V vs RE at a scan rate of 50 mV s^–1^.

The analytical performance of the proposed Re-SPCEs was evaluated by employing CV experiments. For constructing the analytical curve for DOP, CV measurements were performed in phosphate buffer solution (PB, 0.1 mol L^–1^, pH 7.0) within the potential range of −0.4 to 0.6 V at a scan rate of 100 mV s^–1^. DOP solutions with concentrations of 10, 20, 40, 60, 80, 120, and 160 μmol L^–1^ were analyzed. To construct the analytical curve for TRP, CV experiments were performed in Britton–Robertson buffer solution (BR, 0.2 mol L^–1^, pH 2.0) within the potential range of 0.2 to 1.2 V at a scan rate of 100 mV s^–1^. The utilized concentrations of TRP were 10, 25, 50, 75, 100, 150, and 200 μmol L^–1^. The values of the LODs were calculated using the equation LOD = 3*S*/*b*, where *S* represents the standard error of the intercept, and *b* is the slope of the calibration curve.^32^

Repeatability and reproducibility studies were also performed for both analytes, DOP and TRP. The repeatability assay was assessed using a Re-SPCE and analyte concentrations of 80 and 100 μmol L^–1^ for DOP and TRP, respectively. For conducting the reproducibility study, levels of 80 μmol L^–1^ (DOP) and 25 μmol L^–1^ (TRP) were employed. Each electrochemical experiment was conducted using a sample aliquot of 100 μL in triplicate and at room temperature (25 ± 2 °C).

### 2.5 Sample Analysis

To realize the recovery study involving the method for DOP, artificial urine samples were spiked with DOP at three different concentrations (10, 50, and 80 μmol L^–1^). For this experiment, solutions were prepared at a ratio of 1:100 (standard solution: artificial urine—Surine). Regarding the analysis of TRP, a tryptophan capsule from Catarinense Pharma (containing 430 mg of the active ingredient) was dissolved in 250 mL of ultrapure water. Next, a sample aliquot of 40 μL was diluted with 960 μL of ultrapure water. For performing the analysis of TRP, a sample aliquot (20 μL) was further diluted in 980 μL of supporting electrolyte (0.2 mol L^–1^ B–R buffer, pH 2.0). After completing the desired analysis, the ecological profile of the proposed method was calculated employing the AGREE method.^33^

### 2.6 Characterization

Electrochemical impedance spectroscopy (EIS) analysis was performed using a Dropsens workstation (Herisau, CH) managed by DropView 8400 M software. Surface morphological characterization was performed by scanning electron microscopy (SEM) using a JEOL JSM-6390LV microscope (Akishima, TYO, JP) operating at 30.0 kV. Water contact angle measurements were carried out by a Ramé-Hart 250-F1.

EIS measurements were carried out using frequencies ranging from 100 kHz to 0.01 Hz (potential amplitude of 10 mV). The experiments were performed for freshly purchased SPCEs and Re-SPCEs in 1.0 mmol L^–1^ [Fe­(CN)_6_]^3–/4–^ prepared in 0.1 mol L^–1^ KCl.

The morphological study by SEM was carried out by capturing images of the WE surface of an SPCE, a discarded SPCE, and an Re-SPCE. Contact angle (CA) measurements were performed by using distilled water droplets (5 μL) placed on the surface of the WE.

## 3 Results and Discussion

### 3.1 Fabrication of the Re-SPCE

Despite their widespread use in electroanalytical applications, one probable limitation of commercial SPCEs lies in their classification as disposable devices.^34,35^ This nature can be particularly problematic in redox activities involving strongly adsorbing analytes, which often compromise signal repeatability and long-term utility, in some cases.^36,37^ To overcome this limitation, the present work collected unusable SPCEs and utilized a combination of a self-adhesive mask and homemade carbon-based ink to cover the electrodes again.

A low-cost craft cutter printer of ∼550.0 USD was employed to create the stencil mask. The conductive ink was prepared following procedures previously described in the literature.^38^ The proposed protocol for recycling the SPCEs can be detailed in five steps: (i) collection of discarded SPCEs; (ii) cutting of the self-adhesive stencil mask according to the electrode dimensions and attachment onto the device surface; (iii) deposition of the conductive ink; (iv) removal of the self-adhesive mask; and (v) Re-SPCEs ready for use. One batch of carbon-based ink can be used to create ∼36 electrodes. The calculated device cost, in terms of consumables materials, was ∼0.013 USD (Table S1).

It is important to mention that the SPCE, especially model DRP-110, has ceramic substrates that bring suitable mechanical performance and chemical stability. Also, in this model of SPCE, there is Ag ink in all electrical contacts, a noble metal, which is another great reason for the metal to be recycled.

Other recovery strategies have also been extensively explored by the scientific community. For instance, Jin and Zhang^39^ proposed a sustainable process for recovering metals from waste streams, while Dutta et al.^40^ developed a recycling approach for printed circuit boards, emphasizing environmentally responsible metal recovery. More recently, Caldas et al.^41^ demonstrated the reuse of discarded plastic cups as raw materials for producing 3D printing filaments, which were subsequently employed in the fabrication of electrochemical sensors. Similarly, Spirio et al.^42^ reported the valorization of plastic residues from discarded electronic equipment, mainly talc-filled polypropylene, into functional 3D printing filaments, highlighting a circular approach to waste utilization.

### 3.2 Morphology Characterization

The surface morphology of the electrodes was investigated using SEM. Images were acquired from the WE surface from three different sensors: (i) as-purchased SPCE, (ii) discarded SPCE, and (iii) Re-SPCE, as illustrated in Figure 2.

**Figure 2:**
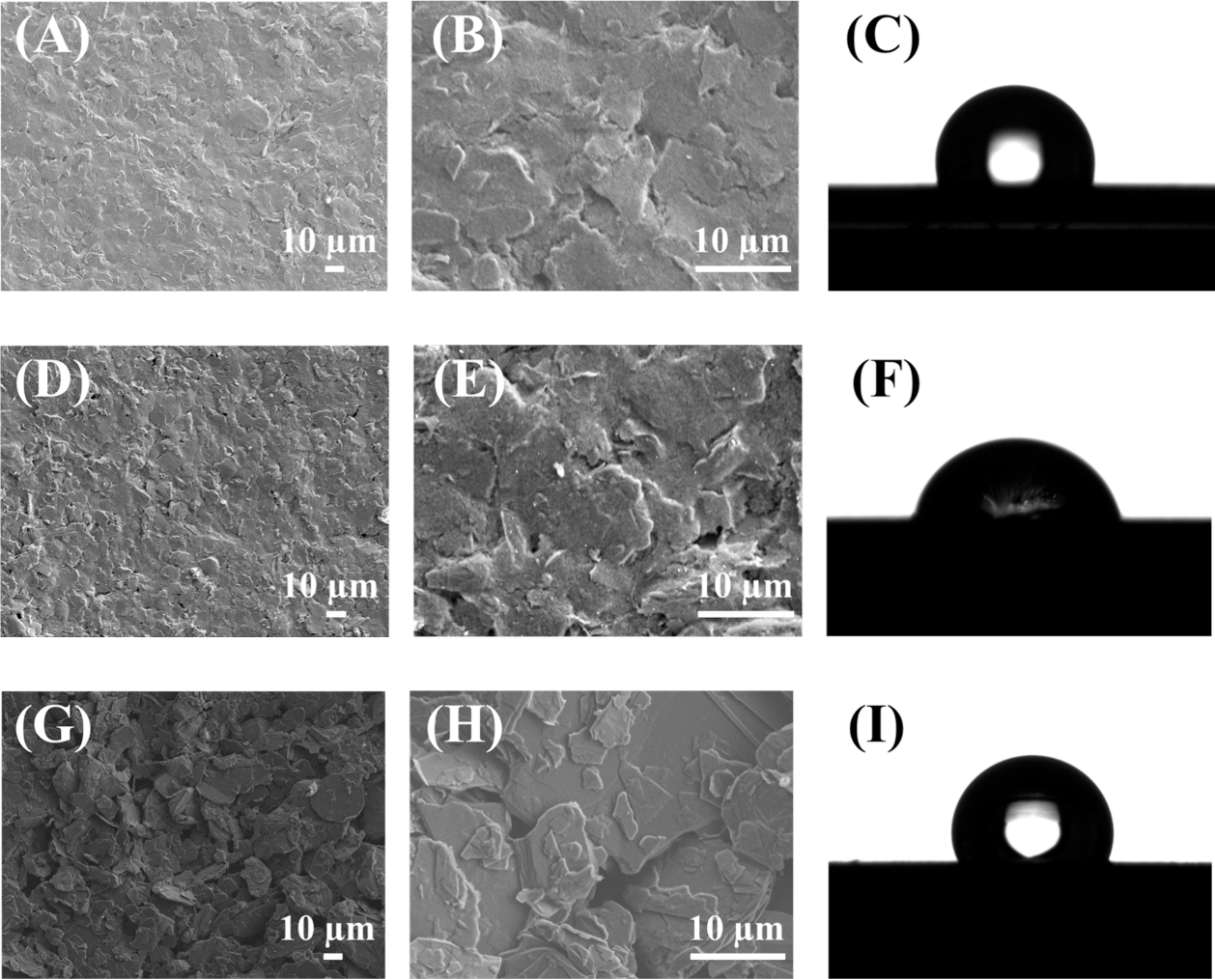
Obtained SEM images and CA results for the WE integrated on SPCE: (A and B) SPCE at magnifications of ×500 and ×2500, respectively; (D and E) discarded SPCE at ×500 and ×2500; (G and H) Re-SPCE at ×500 and ×2500. CA images of (C) SPCE, (F) discarded SPCE, and (I) Re-SPCE.

From Figure 2A, it is possible to see that the native WE (i) exhibited a well-homogeneous surface successfully incorporated with carbon particles, as traditionally observed for commercial SPCEs.^2^ However, a drastic difference on the WE surface was verified for sample (ii), probably associated with an excessive number of scans and the adsorption of molecules. Maintaining the integrity of the WE surface is essential for ensuring accurate and reproducible electrochemical measurements. However, the application of potential during experiments can induce morphological changes, such as increased roughness, as illustrated in Figure 2D,E, which compares a new electrode with one subjected to repeated use. These modifications are typically associated with electrochemical stress and mechanical wear.

On the other hand, the WE sample from the Re-SPCE displayed a surface morphology that was less compact than the typically dense and uniform structure when compared to the as-purchased WE surface (Figure 2G,H). Interestingly, the increased roughness observed in the Re-SPCE is not necessarily disadvantageous. Previous studies have shown that surface activation of printed electrodes, often achieved by inducing roughness, can enhance the electroactive surface area and consequently improve electrochemical performance.^43^

CA measurements were also performed to evaluate the wettability of the electrode surfaces. Figure 2C,F,I shows the obtained results for the as-purchased SPCE, discarded SPCE, and Re-SPCE, each measured in triplicate. The SPCE exhibited a CA of 105 ± 2°, indicating a hydrophobic surface. In contrast, the discarded electrodes displayed a lower and more variable CA (95 ± 15°), likely reflecting differences in usage history. Binder materials are incorporated to secure the carbon layer to the underlying silver contact. After repetitive experiments, these binders can leach, leading to surface alterations.^44^ As shown in Figure 2I, the Re-SPCE exhibited a CA of 108 ± 3°. We believed that despite the addition of a varnish-based binder during the recovery process, its hydrophobicity ability remained comparable to the as-purchased WE surface from the SPCE.

### 3.3 Optimization of the Reference Electrode

During prolonged use, significant alterations can be observed on the RE surface, which are mainly associated with silver oxidation. Differences among the discarded electrodes are evident, reflecting their distinct usage histories and the resulting extent of silver oxidation in the RE (Figure S2). This deterioration is likely intensified by prolonged exposure to highly acidic or alkaline electrolytes, which can accelerate silver corrosion.^45^ To address this issue, a comparative study was performed employing three different options of the RE: (i) saturated Ag/AgCl_sat_, (ii) native Ag, and (iii) carbon ink. CV experiments were performed in the absence and presence of 1.0 mmol L^–1^ [Fe­(CN)_6_]^3–/4–^ (Figure S3).

As shown in Figure S3A, when utilizing the Re-SPCE with Ag/AgCl_sat_ as the RE, well-defined cathodic and anodic peak potentials were observed at 0.39 and −0.09 V, respectively. From Figure S3B, it is possible to inform that after recording 10 scans, in sequence, the calculated values of standard deviation (SD) were 2.70 and 3.16% involving the anodic and cathodic peak currents (*I*_pa_ and *I*_pc_), respectively.

When performing CV experiments by using REs composed of Ag (Figure S3C,D) and carbon (Figure S3E,F), cathodic and anodic peak potentials for [Fe­(CN)_6_]^3–/4–^ were also observed. The Re-SPCE with Ag ink, as the RE, has exhibited a shift potential of 250 mV when compared to that with Ag/AgCl_sat_. A shift potential of ∼290 mV was calculated for the same redox probe ([Fe­(CN)_6_]^3–/4–^) when employing the Re-SPCE with carbon ink as the RE. The abovementioned results have indicated that carbon-based reference electrodes exhibit potential shifts due to the absence of a fixed redox couple, making their potential highly dependent on surface states and ionic composition of the solution.^45–47^

The repeatability study of the RE composed of Ag and carbon inks was also evaluated. The obtained value of SD for the oxidation peak potential decreased from 7.70% (Ag ink as the RE) to 2.12% (carbon ink as the RE), and for the reduction peak, it dropped from 13.50 to 0.78%. These results have demonstrated that Ag ink and carbon ink can be utilized as REs. To reduce the cost associated with the fabrication of the RE, all subsequent electrochemical experiments were conducted using Re-SPCEs, in which the RE was coated with carbon ink. This option of configuration has provided greater repeatability and negligible potential drift, making it a reliable alternative to the RE, as also observed for the silver-based reference system that is utilized in as-purchased SPCEs.

### 3.4 Electrochemical Characterization

EIS was employed to obtain a deeper understanding of the interfacial electrochemical behavior and charge-transfer characteristics of the electrode surface. Figure S4 presents the Nyquist plots obtained by using 1.0 mmol L^–1^[Fe­(CN)_6_]^3–/4–^ prepared in 0.1 mol L^–1^ KCl. Measurements were carried out for the SPCE and Re-SPCE. The calculated values of charge-transfer resistance (*R*_ct_) for the SPCE and Re-SPCE were 4.68 and 25.20 kΩ, respectively. The obtained result of *R*_ct_ for the Re-SPCE has exhibited a 5.3-fold higher when compared to the SPCE. This behavior less conductive for the proposed WE can be attributed to the utilized carbon-based ink, which is composed of other sources of conductive material (graphite flakes) and a binder (glass varnish).^48–50^

Figure 3 presents the CVs obtained for the SPCE and Re-SPCE, recorded in a solution containing 1.0 mmol L^–1^ [Fe­(CN)_6_]^3–/4–^ prepared in 0.1 mol L^–1^ KCl. The experiments were conducted under varying scan rates ranging from 10 to 200 mV s^–1^. The Re-SPCE used in this experiment retained the native reference electrode (Ag) of the commercial SPCE, thus avoiding the potential difference observed in the previous study.

**Figure 3:**
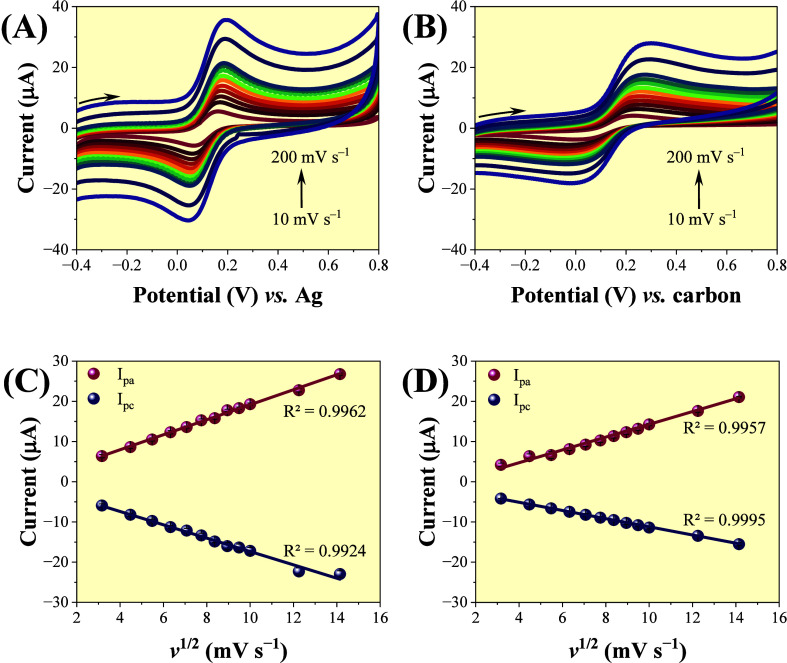
CVs recorded for 1.0 mmol L^–1^ [Fe­(CN)_6_]^3–/4–^ in 0.1 mol L^–1^ KCl using (A) SPCE (B) Re-SPCE with the variation of the scan rate from 10–200 mV s^–1^; peak current vs square root of speed (*v* ^1/2^) for (C) SPCE and (D) Re-SPCE.

A comparison of Figure 3A,B revealed some differences between the two electrochemical systems. The main distinction was related to peak intensity, with the SPCE exhibiting more pronounced oxidation and reduction peaks (Figure 3A), which can be attributed to a smoother and more uniform surface, resulting in lower charge-transfer resistance. In contrast, the CVs obtained with the Re-SPCE (Figure 3B) showed slightly lower *I*_p_, broadened peaks, and greater peak-to-peak separation (Δ*E*_p_). At a scan rate of 100 mV s^–1^, the current intensities were 19.5 μA for the SPCE and 14.3 μA for the Re-SPCE. As highlighted by Kadara et al.,^2^ tests performed on different options of commercial electrodes demonstrate that several factors can influence catalytic ability negatively. The reason why the Δ*E*_p_ values of various strips often deviate from the ideal is not straightforward and may be attributed to multiple factors, including (i) the type of graphite used in the ink, particularly the proportion of edge-plane sites;^51–53^ (ii) graphite loading; (iii) surface functionalization and the presence of oxygenated groups;^54–57^ (iv) the amount of polymeric binder, which may hinder electron transfer;^58–60^ and (v) differences in surface wettability.^61,62^

It is worth noting, however, that the redox responses of the Re-SPCE remained comparable to those of the SPCE, indicating that the recovered electrodes preserved satisfactory electrochemical activity and efficient redox behavior of the [Fe­(CN)_6_]^3–/4–^ probe.

Figure 3C,D illustrates the linear relationship between *I*_p_ and the square root of the scan rate (*v* ^1/2^) for both electrodes. This dependence is characteristic of diffusion-controlled processes, as described by the Randles–Sevcik model, and reinforces the notion that the rate-limiting step in the electrochemical response is the mass transfer of the analyte from the solution to the electrode/solution interface.^63,64^ These findings corroborate the reversibility of the Fe­(II)/Fe­(III) redox couple on both platforms and demonstrate the robustness of the system for comparative electroanalytical performance studies.

Based on the Randles–Sevcik model, which applies to reversible electrochemical systems under diffusion control,^65^ it was possible to quantitatively estimate the electroactive surface areas of the SPCE and Re-SPCE sensors using the following equation:

$$i_{p}=(2.69\times 10^{5})n^{3/2}A{D_{o}}^{1/2}C_{o}v^{1/2}$$

where *I*_p_ is the peak current (A), *n* is the number of electrons transferred (*n* = 1), *A* is the electroactive area (cm^2^), *D*_o_ is the diffusion coefficient of potassium ferricyanide (6.39 × 10^–6^ cm^2^ s^–1^), *C*_o_ is the analyte concentration (mol cm^–3^), and *v* is the scan rate (V s^–1^). Based on this equation and the obtained redox results, the calculated electroactive areas were 0.062 cm^2^ for the Re-SPCE and 0.091 cm^2^ for the SPCE. These values reflect differences in the active surfaces of the electrodes and are consistent with the voltammetric observations previously discussed.

Additionally, the heterogeneous electron transfer rate constant (*K*_s_) was calculated for the Re-SPCE and SPCE.^67^ The Re-SPCE exhibited a *K*_s_ value of 1.2 × 10^–4^ cm s^–1^, while the SPCE presented a *K*_s_ value of 6.1 × 10^–4^ cm s^–1^. The lower value obtained for the Re-SPCE, when compared to the SPCE, can be attributed to the utilization of another source of graphite flakes on the conductive ink, and this behavior agree with the catalytic differences previously observed in the presence of [Fe­(CN)_6_]^3–/4–^.^66,67^

### 3.5 Repeatability and Reproducibility

The repeatability study of the Re-SPCE was evaluated using 1.0 mmol L^–1^ of [Fe­(CN)_6_]^3–/4–^ prepared in 0.1 mol L^–1^ of KCl. In this experiment, 10 consecutive measurements were performed. Also, the performance of the proposed electrode was compared to that of an SPCE (Figure 4).

**Figure 4:**
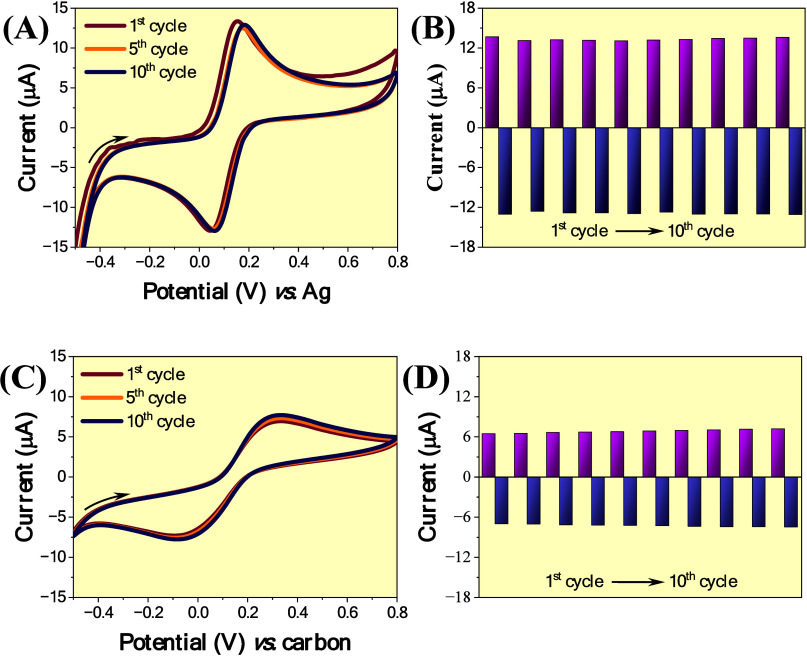
CVs obtained for the repeatability study using (A) SPCE and (C) Re-SPCE at a scan rate of 100 mV s^–1^. Corresponding *I*_p_ values from 10 independent scans for (B) SPCE and (D) Re-SPCE.

When considering the SPCEs only, the calculated values of relative standard deviation (RSD) were 1.6 and 1.2% for the *I*_pa_ and *I*_pc_ signals, respectively (*n* = 10), confirming excellent repeatability. For the cases when the Re-SPCE was employed, the results of RSD associated with *I*_pa_ and *I*_pc_ were found to be ∼3.7 and 2.3%, respectively (*n* = 10), indicating similar quality when compared to the as-purchased SPCE.

The reproducibility analysis was evaluated by employing 16 different sensors. In this experiment, electrochemical measurements were performed on the electrodes before and after the recovery process. Figure S5 shows the CVs of all tested electrodes, while Figure 5A,B presents the CVs of three randomly selected discarded SPCEs and those of the same electrodes after recovery, respectively.

**Figure 5:**
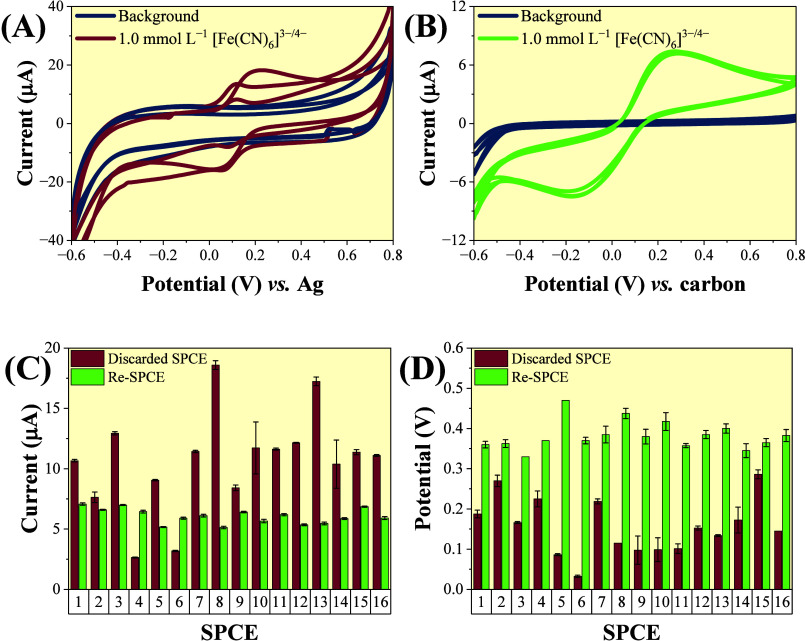
Recorded CVs in 1.0 mmol L^–1^ [Fe­(CN)_6_]^3–/4–^ prepared in 0.1 mol L^–1^ KCl at a scan rate of 50.0 mV s^–1^ for three (A) discarded SPCEs and for three (B) freshly Re-SPCEs. Obtained histograms summarizing the values of (C) *I*_pa_ and (D) Δ*E*_p_ for 16 different sensors utilized before and after the recovery process.

The results evidenced the irreproducibility among the discarded electrodes, as each one had previously been used for a different purpose, leading to inconsistent electrochemical behaviors. After recovering, the Re-SPCE exhibited a reproducible response, displaying consistent CV profiles in the presence of [Fe­(CN)_6_]^3–/4–^ (Figure 5B).

When considering the *I*_pa_ and Δ*E*_p_ values of 16 different sensors over four consecutive measurements, as shown in Figure 5C,D. The obtained histogram, referring to the I_pa_ value, exhibited fluctuations with an RSD value of ∼10.3% for Re-SPCEs when compared to ∼38.7% of RSD for discarded SPCEs (Figure 5C). The signal instability observed for the discarded electrodes can be associated with differences in usage history, where factors such as cleaning procedures, analyte exposure, possible surface modifications, and overall time of use cannot be strictly controlled. In contrast, the Re-SPCEs exhibited stable peak current responses for [Fe­(CN)_6_]^3–/4–^, confirming that the unusable SPCE can be recovered and utilized as a catalytic material again.

Figure 5D exhibits the evaluation of Δ*E*_p_ between the anodic and cathodic peaks of the redox probe. As observed previously, the discarded electrodes exhibited substantial variability in Δ*E*_p_, with an RSD of 44.5%, whereas the recovered electrodes demonstrated greater stability and consistency, showing a much lower RSD of 9.2% (*n* = 16).

### 3.6 Analytical Performance

The Re-SPCE was utilized in association with the CV method for constructing the analytical curves. The molecules DOP and TRP were employed as target analytes. Concentrations ranging from 10 to 160 μmol L^–1^ were utilized for DOP (Figure 6A). The obtained analytical curve (Figure 6B) was described by the equation: *I*_p_ (μA) = 0.04148 [DOP (μmol L^–1^)] + 0.12672, with a determination coefficient (*R* ^2^ = 0.9983). The limit of detection (LOD) was estimated to be 3.7 μmol L^–1^.

**Figure 6:**
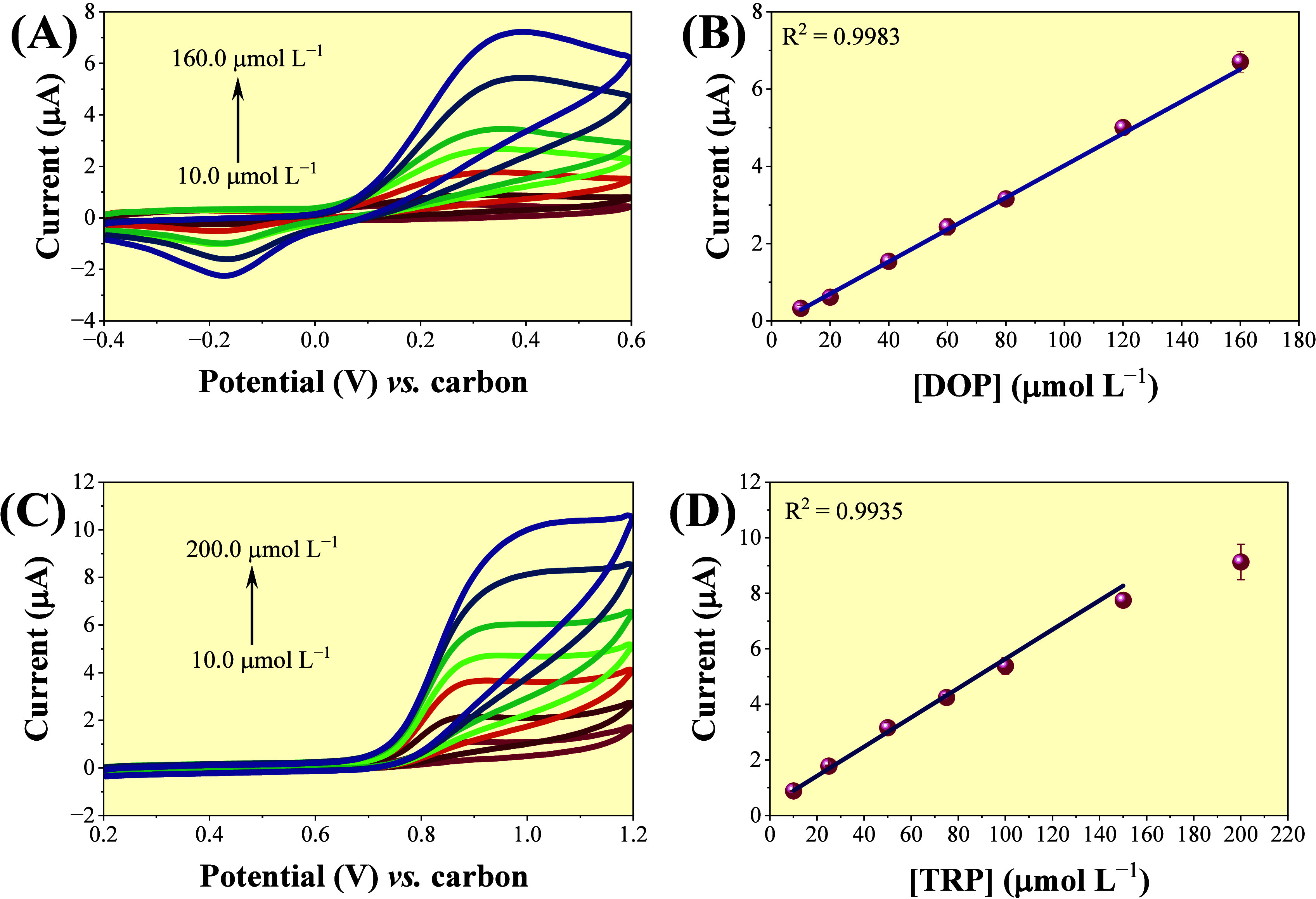
(A) Obtained CVs of DOP at a scan rate of 100 mV s^–1^ and different concentrations prepared in phosphate buffer. (B) Analytical curve of DOP employing concentrations between 10 and 160 μmol L^–1^. (C) CVs of TRP at a scan rate of 100 mV s^–1^ and different concentrations recorded in B–R buffer. (D) Analytical curve of TRP using concentrations ranging from 10 to 150 μmol L^–1^. Each point represents the average of triplicate measurements performed with three different Re-SPCEs.

Similarly, concentrations between 10 and 150 μmol L^–1^ (Figure 6C) were employed to construct the analytical curve of TRP (Figure 6D). The obtained equation for TRP was *I*_p_ (μA) = 0.05267 [TRP (μmol L^–1^)] + 0.37329, with a determination coefficient (*R* ^2^ = 0.9935). The calculated value of the LOD was 2.15 μmol L^–1^.

As comparative experiments, analytical curves were also constructed by using as-purchased SPCEs (Figure S6). The results obtained exhibited slightly higher sensitivities for both analytes. For DOP, the analytical curve was described by *I*_p_ (μA) = 0.04148 [DOP (μmol L^–1^)] + 0.12672, yielding an LOD of 0.98 μmol L^–1^ (Figure S6B). For TRP, the linear regression *I*_p_ (μA) = 0.0861 [TRP (μmol L^–1^)] + 0.3083 resulted in an LOD of 0.91 μmol L^–1^ (Figure S6D). Although the SPCEs provided better values of LOD, the Re-SPCE maintained adequate linearity for both analytes, confirming its electroanalytical suitability.

Some reported studies have demonstrated the use of nonpulsed techniques, such as CV and LSV, in the development of analytical methods. Chelly et al.^68^ explored an SPCE modified with AuNP and utilized LSV to detect DOP, achieving an LOD of 0.2 μmol L^–1^. In another document, Narasimhappa and Ramamurthy^69^ reported the use of an electrochemical sensor in association with the CV technique for TRP sensing, with an LOD of 1.1 μmol L^–1^.

The repeatability and reproducibility studies of the proposed methods were also evaluated, as shown in Figure S7. For performing the detection of DOP, five consecutive measurements were utilized (Figure S7C), and the obtained results showed an RSD of ∼5.8%. On the contrary, the reproducibility experiment using five different Re-SPCEs (Figure S7D) exhibited an RSD value of ∼3.5%. On the other hand, the repeatability (Figure S7G) and reproducibility (Figure S7H) experiments involving the analysis of TRP presented RSD values of ∼3.8 and ∼5.9%, respectively.

### 3.7 Sample Analysis and Ecological Profile

To demonstrate the analytical feasibility of the proposed Re-SPCEs, two different analyses were evaluated. In the first scenario, the detection of DOP in artificial urine was conducted. Before the analysis was performed, the urine samples were spiked with DOP at three different levels (10, 50, and 80 μmol L^–1^). The determination of TRP in a dietary supplement sample was also employed. The obtained CV responses for DOP and TRP are presented in Figures S8 and S9, respectively.

From Table 1, it is possible to see that the analysis of DOP in spiked urine samples showed recovery results ranging from 98.9 to 115.4%. The obtained mass of TRP in the supplementary sample was 414 ± 7 mg, and there is no statistical difference when compared to the labeled value of 430 mg (*t* test; *p* = 0.05). These findings have demonstrated that the proposed Re-SPCE can be utilized as a voltammetric sensor.

**Table 1:** Detected Levels of DOP in Artificial Urine Samples Previously Spiked with Standard Solutions at Three Concentration Levels (*n* = 3)

**spiking level**	**add (μmol L** ^**–1**^ **)**	**found (μmol L** ^**–1**^ **)**	**recovery (%)**	**RSD (%)**
#1	10	11.6	116.9	6.7
#2	50	51.9	103.8	7.8
#3	80	84.7	105.8	3.3

To evaluate the ecological profile of the proposed method, the AGREE tool was employed.^33^ This approach can infer how environmentally friendly a method is by employing a punctuation range from 0.0 to 1.0. The presented analytical methodology (Figure S10 and Table S2) exhibited a suitable score of 0.89, resulting in an ecological procedure.^70–75^ The main factors contributing to this high score include the minimal sample preparation requirement (simple dilution), low sample volume (100 μL), reduced waste generation, and the potential for in situ analysis. The main factor slightly lowering the AGREE result was the use of reagents not entirely derived from renewable sources.

As discussed above, the desired detection has provided a suitable AGREE profile, indicating that the combination of the Re-SPCE and voltammetric technique is environmentally friendly.

## 4 Conclusions

This study presents, for the first time, a sustainable and reproducible methodology for the recovery of disposable screen-printed carbon electrodes (SPCEs), specifically the DRP-110-U75 model, transforming them into reusable voltammetric sensors (Re-SPCEs). The proposed protocol, based on an adhesive mask and a homemade conductive ink, proved to be efficient, low cost (∼0.013 USD per electrode), and aligned with the principles of the circular economy. Electrochemical studies demonstrated that the Re-SPCEs sustain quasi-reversible, diffusion-controlled redox responses, with good repeatability and reproducibility compared to as-purchased SPCEs.

The analytical applicability of the Re-SPCEs was demonstrated for the detection of dopamine and tryptophan, showing excellent linearity (*R* ^2^ ≥ 0.99) and satisfactory recovery in complex matrices, such as artificial urine and dietary supplements. Future studies will be dedicated to recovering more options of commercial printed cells and informing environmental analysis with certified reference analytical techniques to further emphasize that planar sensors, especially those composed of ceramic and noble metals, must be recovered before being considered unusable. The informed methodology was considered environmentally friendly, achieving an AGREE score of 0.89, highlighting its low reagent consumption, waste minimization, and suitability for in situ analyses. These results confirm that the Re-SPCE not only extends the lifetime of disposable electrodes but also provides a reliable, economical, and ecologically responsible alternative for applications in electroanalytical chemistry.

## Supplementary Material





## Notes

Electrochemical characterization, production cost of the electrodes, and selected parameters for the AGREE score (PDF)
Protocol for recovering the SPCE (MP4)
