# Supplementary material for: Self-Adhesive Mask and Homemade Carbon Ink: Turning Unusable Screen-Printed Electrodes into a New Voltammetric Sensor
Source: ACS Omega. 2026 Jan 2;11(2):3558–68. doi: 10.1021/acsomega.5c11328 (PMC12824970; doi:10.1021/acsomega.5c11328)
Supplement: Supplementary file 1 [file ao5c11328_si_001.pdf]

Supplementary information for:

Self-adhesive mask and homemade carbon ink: turning unusable screen-printed electrodes into a new voltammetric sensor

Gabriel Chitolina-Rodrigues, Adriano Rogerio Silva Lima, Duane Bortot, Caio Raphael Vanoni, Cristiane Luisa Jost, and Habdias de Araujo Silva-Neto·

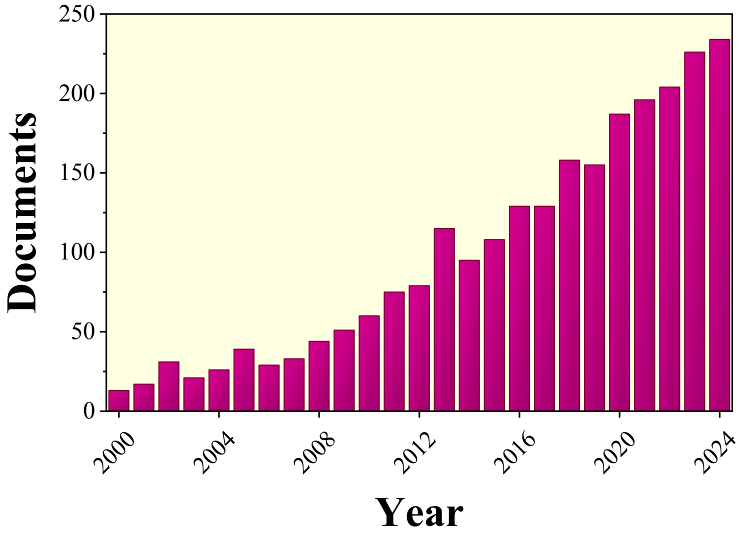

Figure S1. Histogram showing the number of publications retrieved using the keyword "Screen-printed carbon electrode" from 2000 to 2024.

Table S1. Estimated material costs for the preparation of a single batch of Re-SPCEs (Each batch consisted of 36 Re-SPCEs).

Materials used	Quantity per batch	Purchased quantity	Purchase price (USD)	Cost per Batch (USD)
Adhesive vinyl	225 cm^2	30000 cm^2	~ 10	0.075
Soft plastic	225 cm^2	100000 cm^2	~ 6	0.013
Glass varnish	1.5 g	100 g	~ 6	0.09
Graphite powder	1.5 g	500 g	~ 37	0.11
Acetone	4 mL	4000 mL	~ 200	0.20
**Total cost per batch**				**0.48**
**Cost per Re-SPCE**				**0.013**

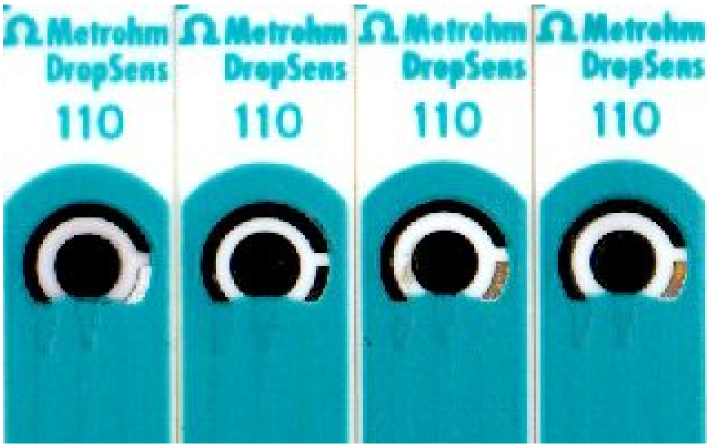

Figure S2. Images of SPCE electrodes: on the left, a commercial SPCE; on the right, three discarded SPCEs exhibiting oxidized silver reference electrodes.

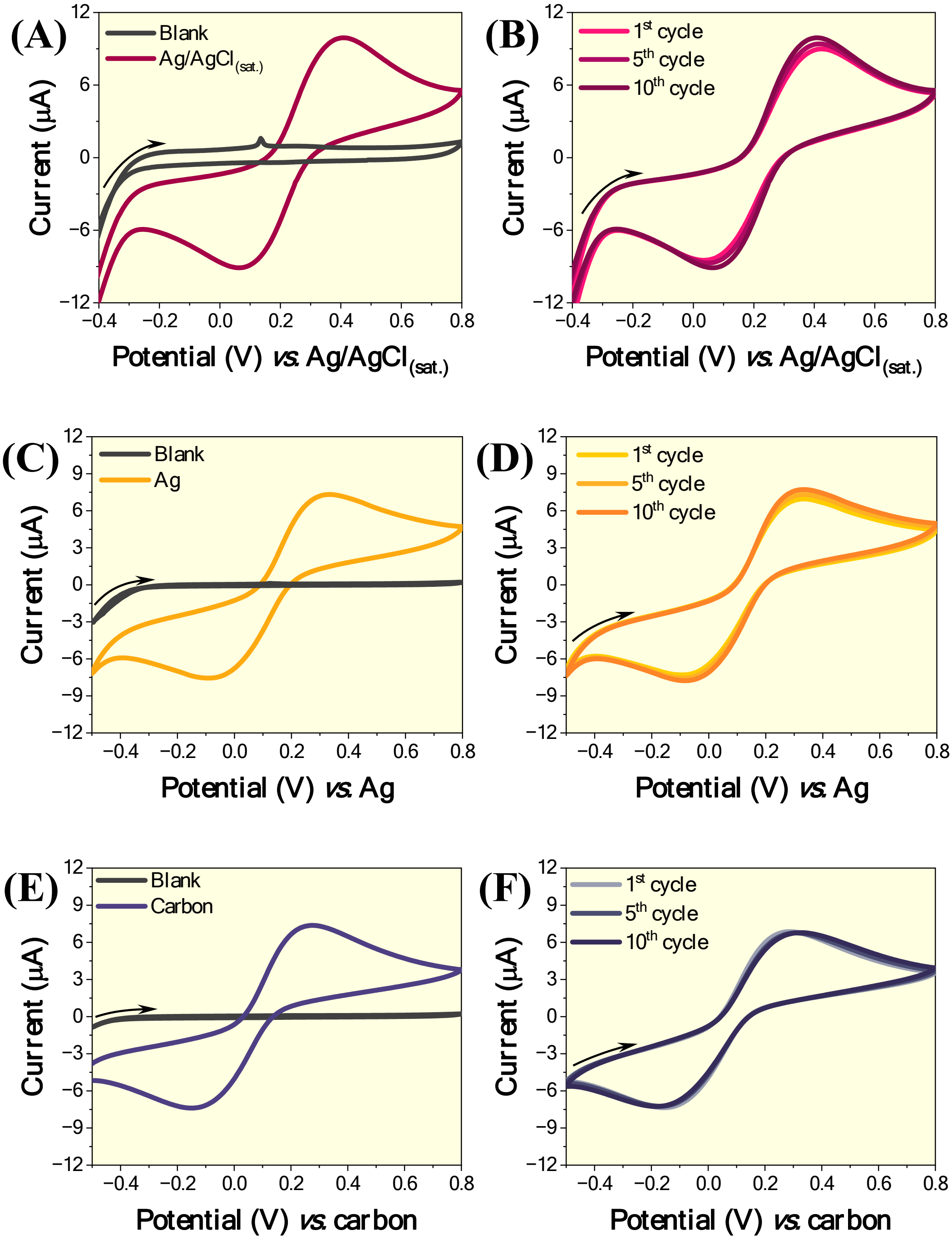

Figure S3. CVs recorded at a scan rate of 50.0 mV s^{-1} using 1.0 mmol L^{-1} $[Fe(CN)_6]^{3-/4-}$ in 0.1 mol L^{-1} KCl solution on the Re-SPCE, employing different reference electrodes: (A) Ag/AgCl$_{sat.}$, (C) silver, and (E) carbon. Repeatability study performed by executing 10 consecutive measurements for each Re-SPCE with the corresponding reference electrodes: (B) Ag/AgCl$_{sat.}$, (D) silver, and (F) carbon.

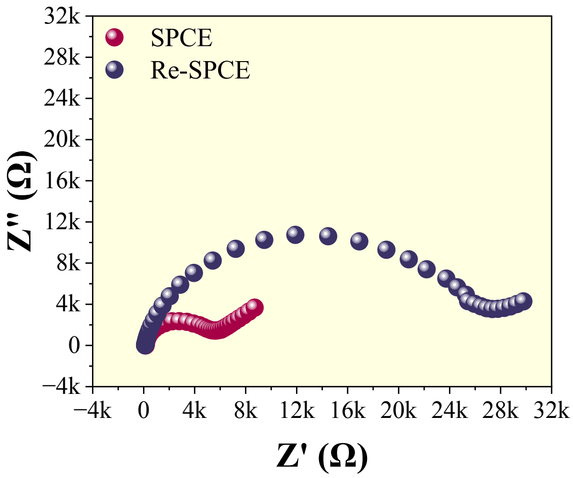

Figure S4. EIS in 1.0 mmol L^{-1} $[Fe(CN)_6]^{3-/4-}$ in 0.1 mol L^{-1} KCl for bare SPCE and Re-SPCE.

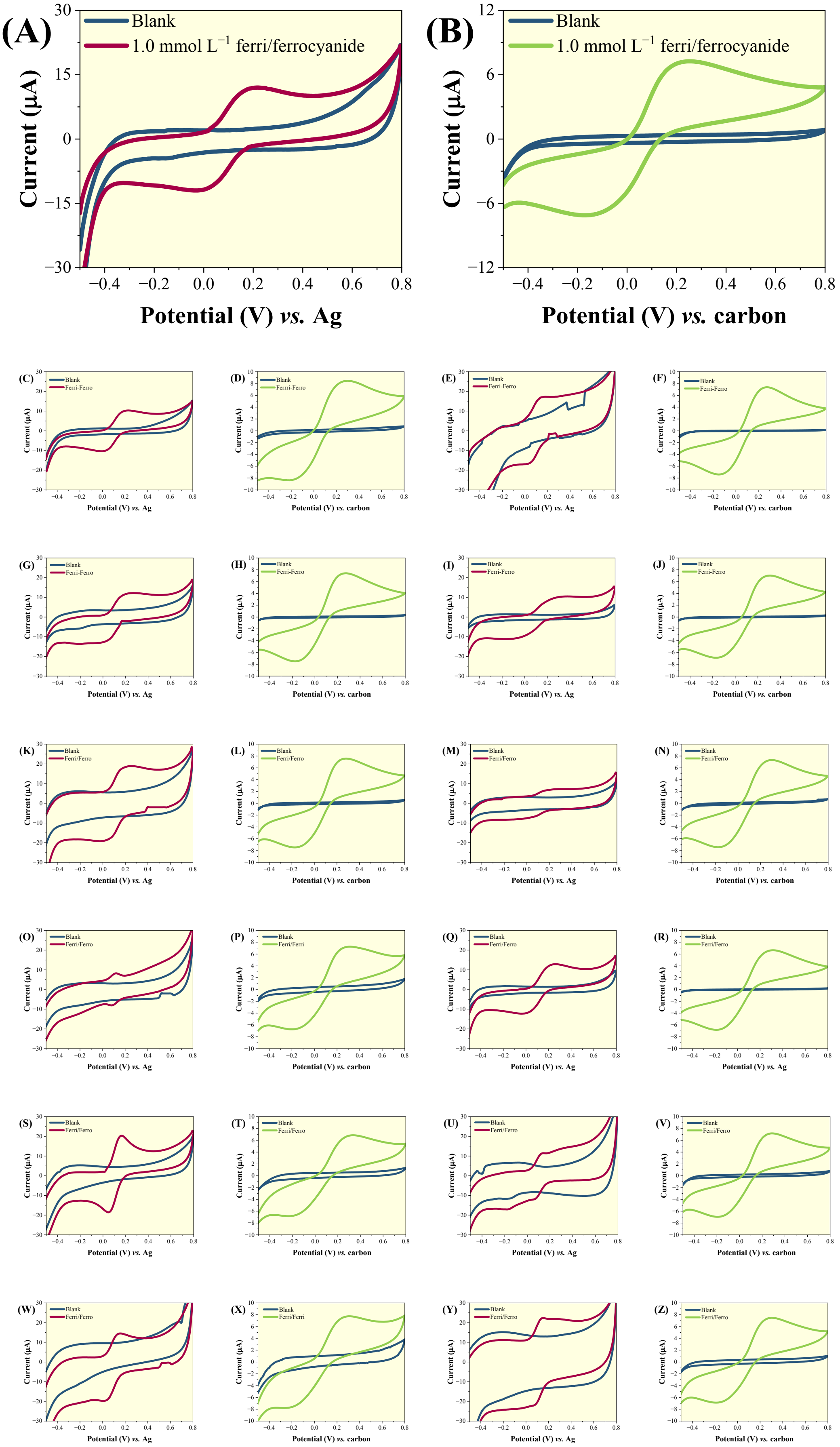
(A)
Blank
1.0 mmol L^{-1} ferri/ferrocyanide
Current (μA)
Potential (V) *vs.* Ag
(B)
Blank
1.0 mmol L^{-1} ferri/ferrocyanide
Current (μA)
Potential (V) *vs.* carbon
(C)
(D)
(E)
(F)
(G)
(H)
(I)
(J)
(K)
(L)
(M)
(N)
(O)
(P)
(Q)
(R)
(S)
(T)
(U)
(V)
(W)
(X)
(Y)
(Z)

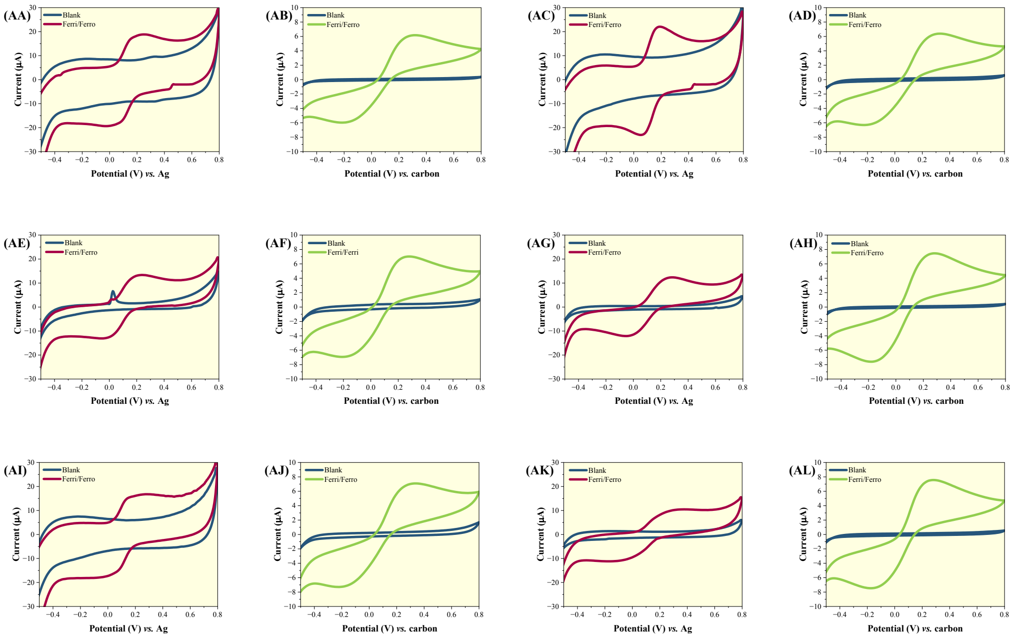

Figure S5. CVs recorded in 1.0 mmol L^{-1} $[Fe(CN)_6]^{3-/4-}$ in 0.1 mol L^{-1} KCl at a scan rate of 50 mV s^{-1} discarded (red) and Re-SPCE (green).

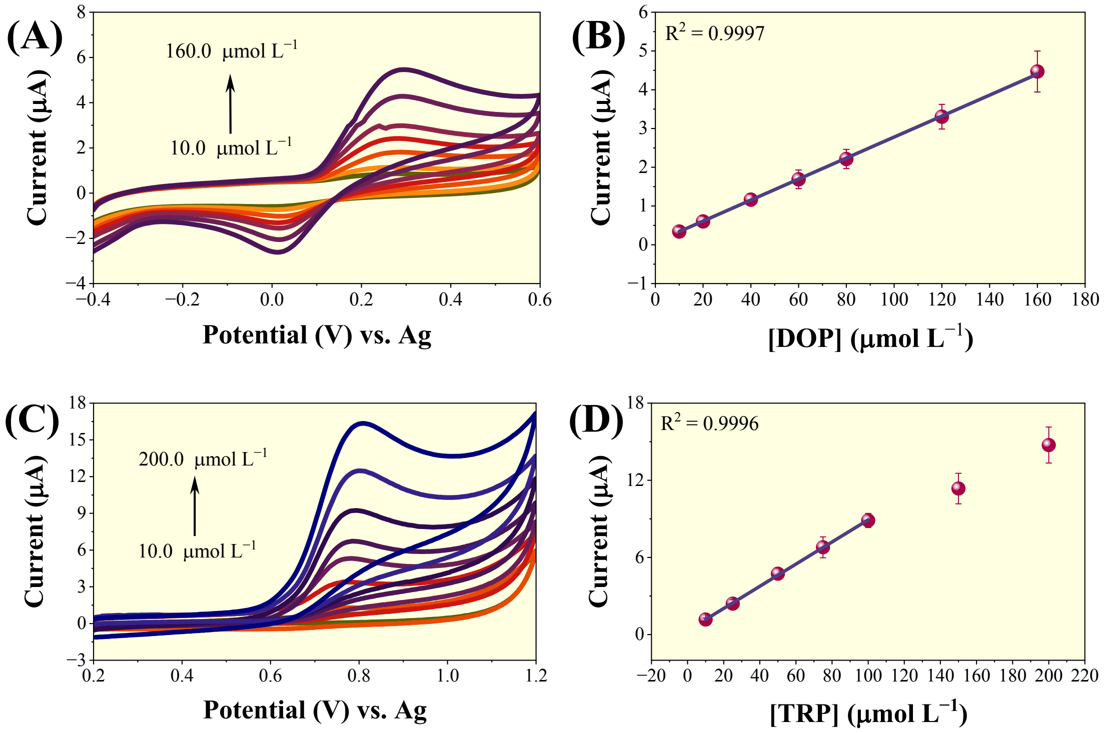

Figure S6. (A) The obtained CVs of DOP at scan rate of 100 mV s^{-1} and different concentrations prepared in phosphate buffer. (B) Analytical curve of DOP employing concentration between 10 and 160 μmol L^{-1}. (C) CVs of TRP at scan rate of 100 mV s^{-1} and different concentrations recorded in B-R buffer. (D) Analytical curve of TRP using concentrations ranging from 10 to 100 μmol L^{-1}. Each point represents the average of triplicate measurements performed with three different new SPCE.

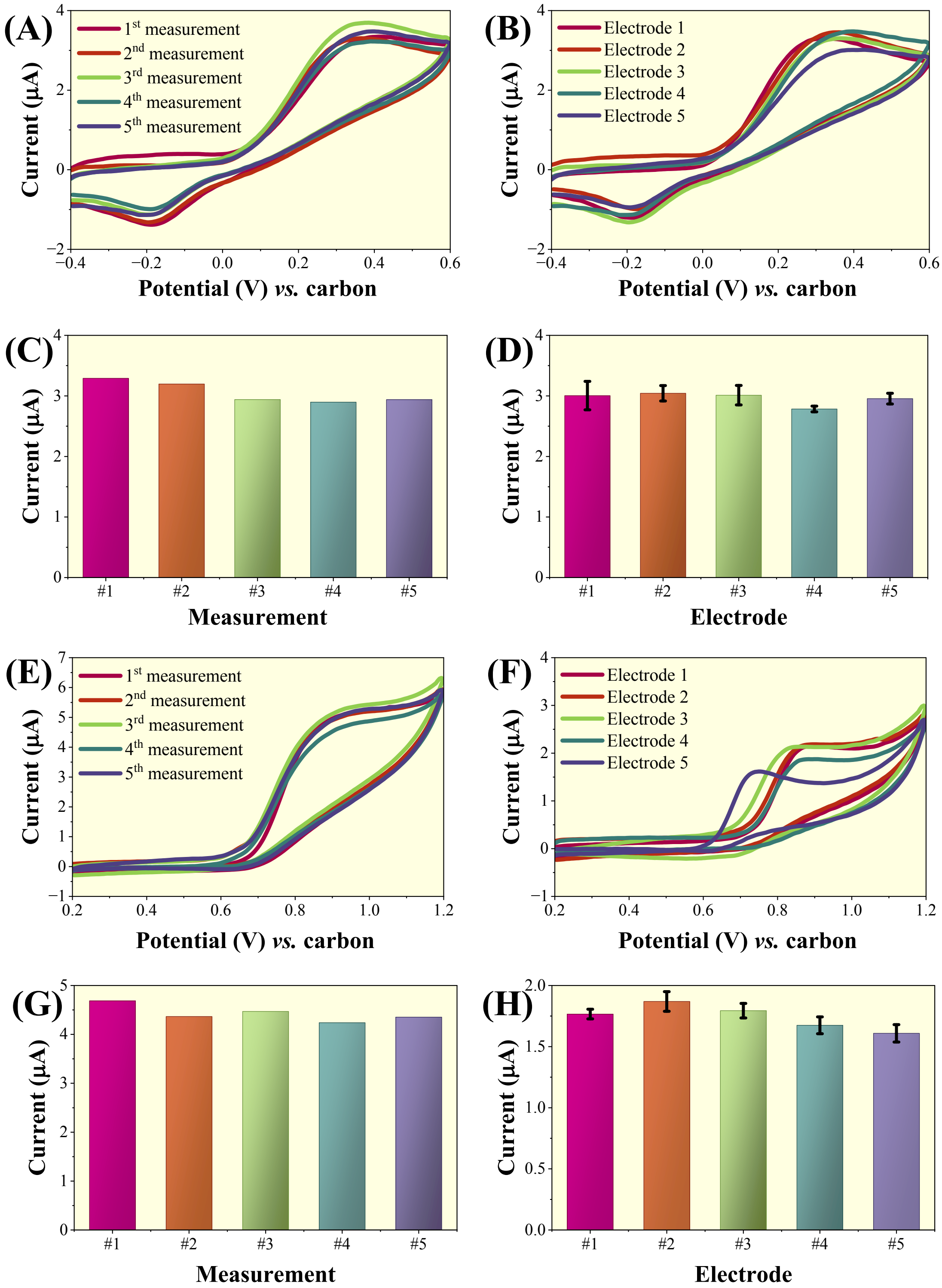

Figure S7. (A) Repeatability study using CV at the same Re-SPCE with DOP at 80 μmol L^{-1}. (B) Reproducibility study using five different Re-SPCEs with DOP at 80 μmol L^{-1}. Scan rate: 100 mV s^{-1}. (C) Histogram of the repeatability study at the same Re-SPCE with DOP at 80 μmol L^{-1}. (D) Histogram of the reproducibility study using five different Re-SPCEs with DOP at 80 μmol L^{-1}. (E) Repeatability study using CV at the same Re-SPCE with TRP at 100 μmol L^{-1}. (F) Reproducibility study using five different Re-SPCEs with TRP at 25 μmol L^{-1}. Scan rate: 100 mV s^{-1}. (G) Histogram of the repeatability study at the same Re-SPCE with TRP at 100 μmol L^{-1}. (H)

Histogram of the reproducibility study using five different Re-SPCEs with TRP at 25 μmol L^{-1}.

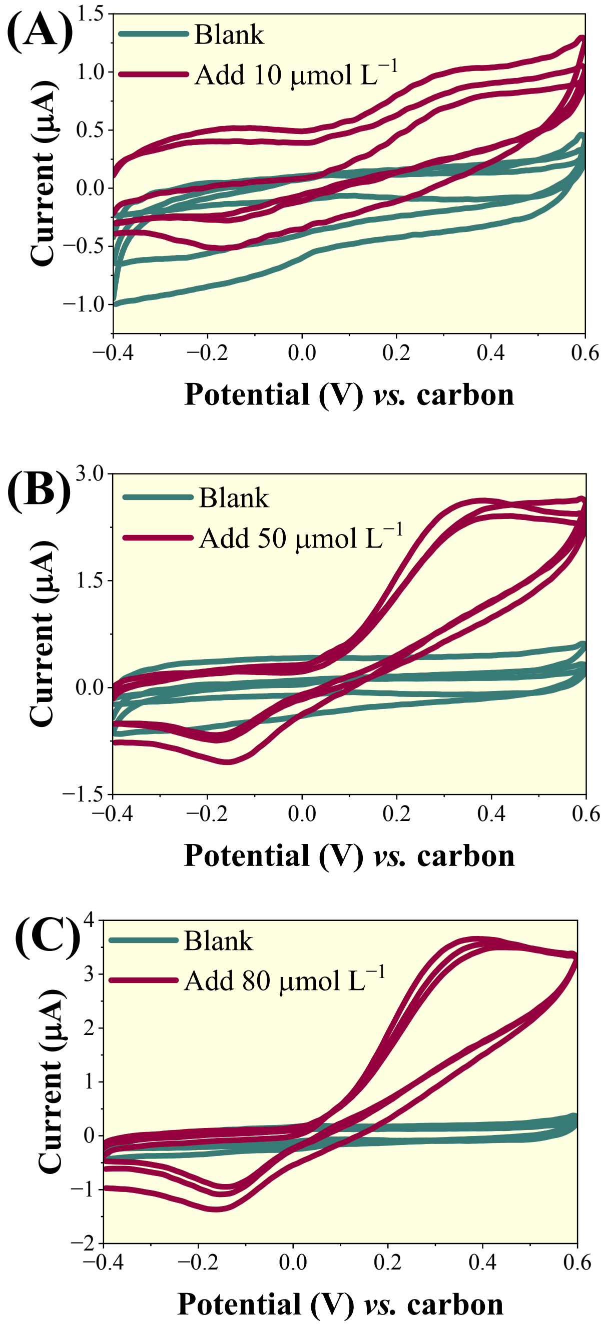

Figure S8. Standard addition study in artificial urine at DOP concentration levels of (A) 10 µmol L^{-1}, (B) 50 µmol L^{-1}, and (C) 80 µmol L^{-1} (n = 3). Experimental conditions: supporting electrolyte, 0.1 mol L^{-1} phosphate buffer (pH 7.0); potential range, −0.4 to 0.6 V; scan rate, 100 mV s^{-1}; artificial urine was diluted 100-fold.

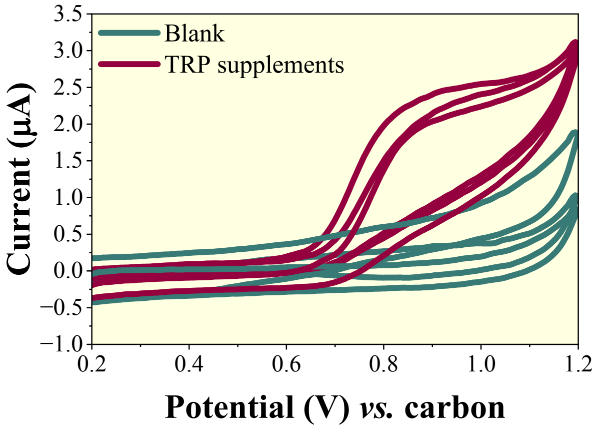

Figure S9. CVs recorded for TRP supplement samples (n = 3). Experimental conditions: supporting electrolyte, 0.1 mol L^{-1} B-R buffer (pH 7.0); potential range, 0.2 to 1.2 V; scan rate, 100 mV s^{-1}.

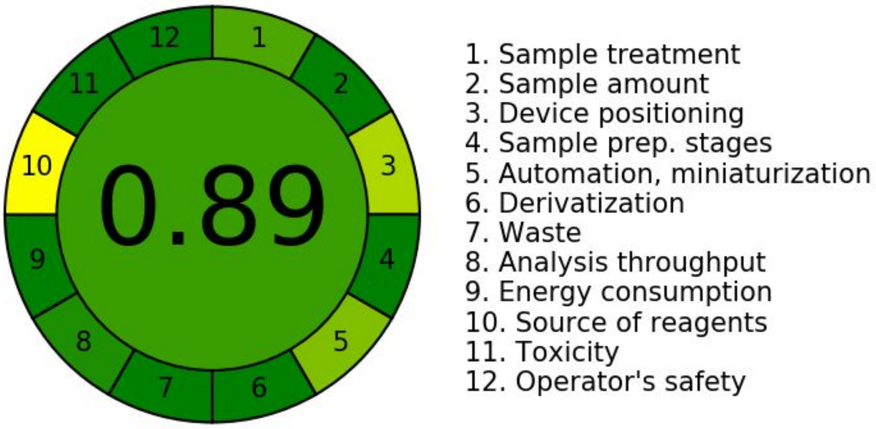

Figure S10. Representative AGREE diagram for the voltammetric determination developed using the Re-SPCE, estimated with AGREE (The Analytical Greenness Calculator).

Table S2. Evaluation of the analytical performance and sustainability criteria for the voltammetric determination using Re-SPCEs, as assessed with the AGREE software.

**Criterion**		**Description/Data**	**Selected classification**
1	Sample treatment	The voltammetric technique, mainly using SPCEs, was introduced to meet the need for in-situ determinations. The method requires minimal sample preparation, typically involving only a simple dilution step.	In-field sampling and direct analysis
2	Sample amount	SPCEs operate with a maximum sample volume of 100 µL, ensuring low sample consumption.	0.100 mL
3	Device positioning	The analysis with the proposed Re-SPCEs is classified as on-line, since sample collection is necessary prior to measurement.	On-line
4	Sample prep. stages	For TRP determination in supplements and DOP in urine, only dilution was required as a sample preparation step.	3 or fewer
5	Automation and miniaturization	Several steps (cleaning, dilution, sample injection) are performed manually; however, detection is automatic. Sample preparation is minimal or miniaturized.	Semi-automatic<br>and<br>None or miniaturized
6	Derivatization	Not applicable to this study.	N/A
7	Waste	Waste generation is minimal, limited to the small sample volume used (~ 100 µL).	0.100 mL
8	Analysis throughput	In this study, only one analyte was determined per run. Each run takes approximately 20 seconds and includes cleaning and sample replacement (~40 s), an average of one analysis per minute can be achieved.	1 per run<br>and<br>60 per hour
9	Energy consumption	The technique uses a potentiostat, consuming approximately 0.005 kWh per analysis.	Potentiostat
10	Source of reagents	The methodology employs some renewable or bio-based reagents.	Some reagents are bio-based

11	Toxicity	The method does not involve the use of toxic reagents in significant quantities.	N/A
12	Operator's safety	No reagents or procedures used pose risks to the operator.	N/A
